# Mood Instability and Irritability as Core Symptoms of Major Depression: An Exploration Using Rasch Analysis

**DOI:** 10.3389/fpsyt.2016.00174

**Published:** 2016-10-26

**Authors:** Lloyd Balbuena, Rudy Bowen, Marilyn Baetz, Steven Marwaha

**Affiliations:** ^1^Psychiatry, University of Saskatchewan, Saskatoon, SK, Canada; ^2^Mental Health and Wellbeing, Warwick Medical School, Coventry, UK

**Keywords:** mood instability, depression, psychometrics, diagnosis, health surveys

## Abstract

**Background:**

Mood instability (MI) and irritability are related to depression but are not considered core symptoms. Instruments typically code clusters of symptoms that are used to define syndromic depression, but the place of MI and irritability has been under-investigated. Whether they are core symptoms can be examined using Rasch analysis.

**Method:**

We used the UK Psychiatric Morbidity Survey 2000 data (*n* = 8,338) to determine whether the nine ICD/DSM symptoms, plus MI and irritability, constitute a valid depression scale. Rasch analysis was used, a method concerned with ensuring that items constitute a robust scale and tests whether the count of symptoms reflects an underlying interval-level measure. Two random samples of 500 were drawn, serving as calibration and validation samples. As part of the analysis, we examined whether the candidate symptoms were unidimensional, followed a Guttman pattern, were locally independent, invariant with respect to age and sex, and reliably distinguished different levels of depression severity.

**Results:**

A subset of five symptoms (*sad, no interest, sleep, cognition, suicidal ideas*) together with *mood instability* and *irritability* satisfactorily fits the Rasch model. However, these seven symptoms do not separate clinically depressed persons from the rest of the population with adequate reliability (Cronbach α = 0.58; Person Separation Index = 0.35), but could serve as a basis for scale development. Likewise, the original nine DSM depression symptoms failed to achieve satisfactory reliability (Cronbach α = 0.67; Person Separation Index = 0.51).

**Limitations:**

The time frame over which symptoms were experienced varied, and some required recall over the last year. Symptoms other than those examined here might also be core depression symptoms.

**Conclusion:**

Mood instability and irritability are candidate core symptoms of the depressive syndrome and should be part of its clinical assessment.

## Introduction

Depression is a common condition with an estimated lifetime prevalence in the USA of about 16% (1). It is an important cause of workdays lost to disability (2) and is as impairing as arthritis, diabetes, and cardiovascular disease (3). The cost of sub-syndromal symptoms probably exceeds that of formally diagnosed major depression (3–6). It is a concern that the incidence of suicide – the most tragic consequence of depression – has not decreased over decades (7). Clearly, we need to better understand the depressive syndrome and the symptoms used in its assessment.

The conceptualization, assessment, and measurement of major depression are tricky, and this shows in the poor reliability and validity of its instruments (8–10). Non-cohesive symptoms might partly explain why specific genetic, biological, or psychological underpinnings are poorly understood (11–13). While depressive symptoms diverge in their association with external variables – as with cognitive and neuro-vegetative symptoms (14) – a particular symptom can be shared by different disorders. For example, it is unclear whether agitation is an indicator of anxiety or depression and whether it is because agitation is related to the higher construct of distress (14, 15). Two individuals can share the same major depression diagnosis without sharing a single symptom (16). Calculating the prevalence and burden of depression is made challenging by the heterogeneity of studies, partly a result of differences in measurement (17).

In clinics worldwide, diagnosing major depression is fairly straightforward. Primary care and specialist physicians follow the DSM (which requires 5 of 9 symptoms) or the ICD (which requires 4 of 10 symptoms) (18). Interestingly, prevalence estimates are similar between systems, although somewhat different populations are identified (19). Having equivalent diagnostic systems has simplified the work of health systems with regard to billing for services and clinical communication (18), but has left important conceptual work unattended. Two problems with the DSM criteria, and by extension, the ICD were raised by Kendler (20). First, the criteria are narrower than the symptoms known to the Western tradition of psychiatry, resulting in an impoverished concept (20). This is perhaps understandable because a list of diagnostic symptoms needs to be brief. Second, the DSM criteria are reified, in the sense that they are thought to constitute depression itself, instead of selected signs of depression (20). In health systems where time is a premium, relying solely on the checklist of symptoms, and ultimately on symptom counts, is a common practice.

Two potential candidate symptoms of the depressive syndrome are MI and irritability. By mood, we mean a valenced emotional state (i.e., positive or negative) (21) in a patient. MI can be defined as “rapid oscillations of intense affect, with a difficulty in regulating these oscillations or their behavioral consequences” (22). MI is closely associated with depression in both cross-sectional and longitudinal studies (23, 24). The prevalence of MI is about 14% in the UK general population and about 61% in participants with depression (25), suggesting that MI could be important in diagnosing depression. MI is central to neuroticism (26) that, in turn, is the personality trait most consistent predictor of depression (27).

DSM-V and ICD-10 accounts of major depression mention irritability in their narrative descriptions, but do not include it in the list of diagnostic symptoms (4, 28). Hence, it could be ignored by clinicians who follow the nine standard symptoms, as if it were an exhaustive list. Yet, it is reported that irritability occurs in one-third to one-half of child and adult patients with major depression (29–31), and is part of a strong principal factor of major depression (29). Irritable depression is also associated with greater severity, lower quality of life, and a history of suicidal attempts, which is itself a criterion for depression (29). These findings, as well as Kendler’s critique suggest that the ICD or DSM symptom lists are incomplete.

Our research questions are:

(i) Do the DSM/ICD symptoms for major depression constitute a valid measure?
(ii) Are MI and irritability symptoms of depression?

We addressed these questions using Rasch analysis, which tests a crucial assumption in scales: the total score (or count of symptoms) is an adequate, equidistant representation of depression levels. In brief, the objective of Rasch modeling is to verify that questionnaires have the properties of physical measures (e.g., a ruler). The units are equally spaced, measure a single attribute (i.e., length), and are additive. Moreover, the reading does not depend on the properties of the entity being measured or the person making the measurement.

## Materials and Methods

### Data

We used data from the 2000 Psychiatric Morbidity Survey (PMS) of Great Britain. The main purpose of the survey was to estimate the prevalence of psychiatric disorders and their correlates using a stratified random multistage design. Participants were 8,580 adults, aged 16–74 years, living in private households in Great Britain. Of these, the 8,338 people (97%) who had complete records of symptoms of interest were the population from which our calibration and verification samples were drawn. Full details of the PMS methods are available in the main survey report (32).

### Measures

#### Depressive Symptoms

Participants were assessed for depression and anxiety disorders by trained lay interviewers who used the Clinical Interview Schedule-Revised (CIS-R). This is a reliable and valid instrument that can be used to algorithmically assign an ICD-10 diagnosis (33). We selected CIS-R questions that were similar in meaning and wording to the nine symptoms of major depression specified by DSM-V. The DSM-V depression symptoms only has “subtle changes” over DSM-IV (34), while both ICD-10 and the upcoming ICD-11 are designed to harmonize with DSM (35, 36). Where the DSM-V symptoms had multiple parts, we combined the participant’s answers to multiple CIS-R questions. The CIS-R questions we included for analysis are the following: “sad, miserable, or depressed,” “unable to enjoy or take an interest in things,” “loss of appetite/weight except on a diet,” “problems getting to sleep/sleeping more than usual,” “restless, walking more slowly, less talkative,” “tired except from doing exercise/lacking in energy,” “felt guilty/blamed self/felt not as good as other people,” “problems concentrating/forgetting things,” “life not worth living/wished for death/thought of suicide.” The time frame over which symptoms were experienced differed for different symptoms (weeks to years), so the duration and timing of occurrence were disregarded.

#### MI and Irritability

These were assessed within the participant-completed Structured Clinical Interview for DSM-IV Axis II Personality Disorders (SCID-II): borderline personality disorder section (37). The question that assessed MI was “Do you have a lot of sudden mood changes?” There were two questions on irritability: (a) “Many people become irritable or short tempered at times, though they may not show it. Have you felt irritable or short tempered with those around you in the past month?” and (b) “During the past month did you get short tempered or angry over things which now seem trivial when you look back on them?”

### Analytical Strategy

**Figure 1:**
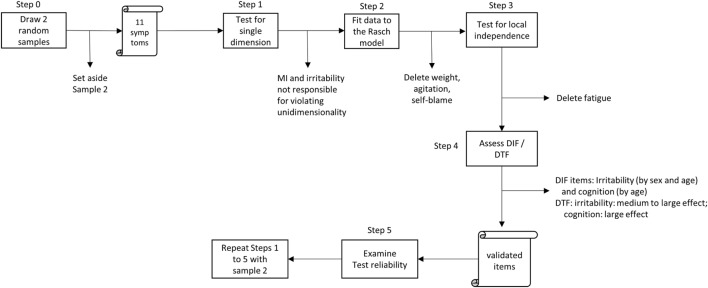
**Schematic diagram of analysis steps**.

Although Rasch analysis is increasingly used in other medical specialties, it is still largely underutilized in psychiatry (38, 39). As mentioned in the Section “Introduction,” Rasch analysis determines whether mental or psychological scales have the characteristics of physical measures. For this to be the case, five conditions must be met (Figure 1). First, the instrument is designed to measure a single attribute (unidimensionality). Just as a ruler measures length only, depression scales must measure depression alone. Second, the responses follow a Guttman pattern: persons are ranked from lowest to highest levels of the trait, while items are ranked by highest to lowest levels of endorsement. The appearance of a Guttman pattern is like a staircase. The ranking of persons and items in this manner produces units that are interval-level measures called logits (“log odds unit”). Third, endorsing a particular question should be independent of the endorsement of another question except with respect to the attribute being measured (local independence). This requirement guards against spurious correlations – those that are due to external factors, such as wording or position in the scale. Fourth, the items and the overall instrument must *not* have differential item or test function. This means that at the question and instrument levels, there must be invariance with respect to person characteristics like age and gender. Fifth, the overall scale must be internally consistent (as measured by Cronbach’s alpha) and able to distinguish different strata of respondents along the latent trait (40, 41).

We performed Rasch analysis in two samples of 500 subjects, one serving as calibration and the other as validation sample. The requirements, tests performed, and the criteria in each test are summarized in Table 1. For the complete description of analysis steps, please refer to the Appendix in Data Sheet S1 in Supplementary Material.

**Table 1:** **Assessment of fit with Rasch model assumptions and the criteria used**.

Requirement		Test(s)	Criterion
1.	Unidimensional	Parallel analysis comparing actual data with artificial data (scope: the entire set of questions) (42)	Retain the *n*th component for which the eigenvalue in the actual data is greater than the 95th percentile in 5,000 simulated data (43)
2.	Guttman pattern	Infit mean square value (scope: per question)	Must be between 0.91 and 1.09 (44)
3.	Local independence	Residual correlations (scope: by pairs of questions)	Not greater than 0.2 and *p* values (with Holm’s adjustment) not greater than 0.05 (45, 46)
4.	Invariance	(a) Uniform DIF: Mantel-Haenszel test (scope: per question) (b) Non-uniform DIF: Breslow-Day (scope: per question) (c) Differential Test Functioning: tau-squared: the variance of DIF across all items (scope: the entire set of questions)	(a)-(b) Neither of the two tests has a significant value (47) (c) tau-squared < 0.07: small DTF 0.07 < tau-squared < 0.14: medium DTF tau-squared > 0.14: large DTF (48)
5.	Reliability	(a) Cronbach alpha (b) Person Separation Index (scope: the entire set of questions)	(a) alpha > 0.8 (b) PSI > 0.7 (41)

## Results

We refer the interested reader elsewhere (32) for a description of the demographic characteristics of all 8,580 PMS participants. Our calibration and verification samples were similar in age distribution (mean = 45 years), mean frequency of depression symptoms endorsed (about four symptoms), sex, and living arrangements. Please refer to Table 2 for details.

**Table 2:** **Demographic variables of the calibration and validation samples taken from the UK Psychiatric Morbidity Survey 2000**.

	Sample 1 (*n* = 500)	Sample 2 (*n* = 500)	*p*
Mean number of symptoms endorsed (of 11 symptoms)	3.83 (2.52)	3.93 (2.46)	0.52
Mean age (SD)	44.48 (15.78)	45.23 (15.64)	0.46
Sex			
Male (%)	213 (42.60)	193 (38.60)	0.20
Female (%)	287 (57.40)	297 (61.40)	
Living arrangements			
Married/cohabiting (%)	273 (54.60)	286 (57.20)	0.70
Single (%)	115 (23.00)	107 (21.40)	
Widowed/divorced/separated (%)	112 (22.40)	107 (21.40)	

### Unidimensionality

Parallel analysis of the 11 candidate items in the calibration sample showed two dimensions (adjusted eigenvalues: 2.82 and 1.02). In the validation sample, a similar result was reached with eigenvalues: 2.84 and 1.04. To determine whether MI and/or irritability were responsible for violating unidimensionality, we omitted them both and performed parallel analysis using only the typical nine depression symptoms. Once again, two dimensions were detected. The eigenvalues were 2.60 and 1.02 for the calibration sample and 2.57 and 1.04 for the validation sample. After misfitting items (i.e., weight/appetite change, agitation/retardation, fatigue, self-blame) were dropped, we tested dimensionality with the retained items (i.e., sad, lack of interest, sleep problems, cognition problems, and suicidal ideas) plus MI and irritability. Only one adjusted eigenvalue was greater than one in both the calibration and validation samples (1.87 and 1.81, respectively). We took these results as evidence that MI and irritability are part of the core indicators of depression.

### Assessment of Fit with a Probabilistic Guttman Pattern

**Figure 2:**
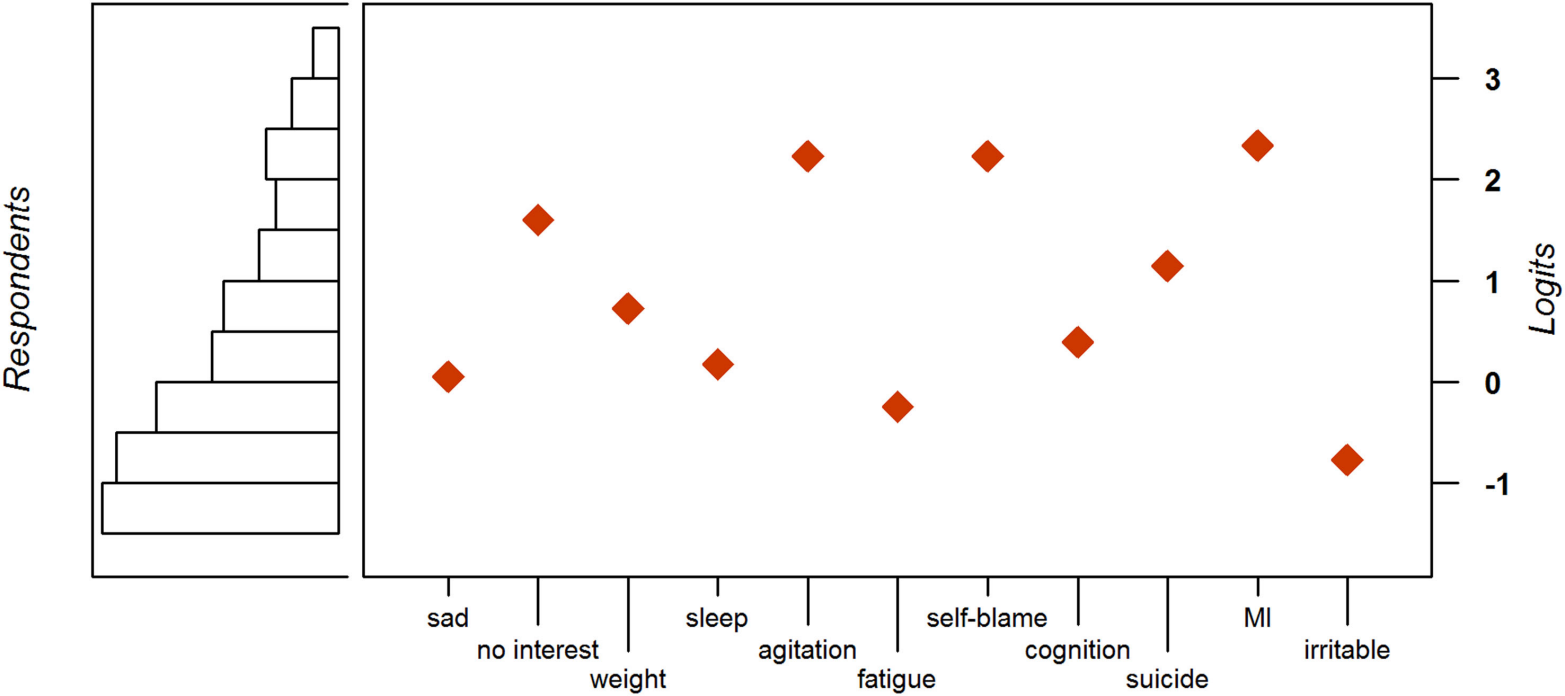
**Distribution of persons and items along the depression latent trait**.

Of the 11 symptoms we examined, the most common symptom was irritability, while MI was the least common. Please refer to Table 3 for the complete list of item locations in logits. Figure 2 is a visual representation of the item locations and the corresponding fraction of the population that they demarcate.

**Table 3:** **Item difficulties (in logits) in the calibration and validation datasets**.

Item	Calibration		Validation	
	Item difficulties in the initial list	Item difficulty after eliminating misfitting items	Item difficulties in the initial list	Item difficulty (after eliminating misfitting items)
1. Sad	0.05	0.06	−0.04	−0.02
2. No interest	1.60	1.57	1.53	1.50
3. Weight/appetite change	0.72	N/A	0.74	N/A
4. Sleep	0.17	0.18	0.14	0.14
5. Agitation, retardation	2.23	N/A	2.00	N/A
6. Fatigue	−0.25	N/A	−0.19	N/A
7. Self-blame	2.23	N/A	2.18	N/A
8. Cognition	0.39	0.39	0.40	0.40
9. Suicidal ideas	1.14	1.13	1.05	1.03
10. MI	2.33	2.29	2.10	2.05
11. Irritability	−0.77	−0.75	−0.96	−0.92

The symptoms that misfit the Rasch model were weight/appetite change, agitation/retardation, fatigue, and self-blame. With consistent findings in both samples, these symptoms were eliminated. See Table 4 for the initial and final symptom lists and fit statistics.

**Table 4:** **Item fit statistics in the calibration and validation datasets**.

Item	Calibration		Validation	
	Infit *t* in the initial list	Infit *t* after eliminating misfitting items	Infit *t* in the initial list	Infit *t* after eliminating misfitting items
1. Sad	0.91	0.93	0.94	0.93
2. No interest	0.97	1.00	1.00	1.04
3. Weight/appetite change	1.19	N/A	1.14	N/A
4. Sleep	1.08	1.07	1.04	1.02
5. Agitation, retardation	0.80	N/A	0.83	N/A
6. Fatigue	0.97	N/A	0.99	N/A
7. Blame no good	0.81	N/A	0.82	N/A
8. Cognition	0.96	0.98	0.97	0.97
9. Suicidal ideas	1.11	1.04	1.10	1.04
10. MI	1.01	0.96	1.00	0.97
11. Irritability	1.05	1.00	1.09	1.03

### Test of Local Independence

In the calibration sample, large residual correlations were observed between the item pairs fatigue and cognition, χ^2^ = 14.36, df = 1, *p* with Holm’s adjustment = 0.004. The validation sample showed a similar large residual correlation for these two items, χ^2^ = 11.26, df = 1, *p* with Holm’s adjustment = 0.02. After fatigue was eliminated from the symptom list, no large residuals remained.

### Differential Item/Test Function

With the assessment of differential response by gender, no items were flagged for DIF in the calibration sample. In the validation sample, irritability showed both uniform and non-uniform effects. Female respondents more frequently endorsed irritability, and the disparity with male respondents also differed by depression level. The impact of DIF by gender with respect to irritability had a medium sized effect on the test for the validation sample. See Table 5 for details.

**Table 5:** **DIF by gender**.

Item	Sample 1 (*n* = 500)					Sample 2 (*n* = 500)				
	Mantel–Haenszel χ^2^^♣^	*p-*Value	Breslow-Day^†^	*p-*Value	Combined decision rule*	Mantel–Haenszel χ^2^^♣^	*p-*Value	Breslow-Day^†^	*p-*Value	Combined decision rule*
Sad	0.85	0.36	5.34	0.25	Ok	1.17	0.28	4.00	0.55	Ok
No interest	0.03	0.85	0.65	0.99	Ok	0.00	0.97	2.39	0.79	Ok
Sleep	1.82	0.18	7.54	0.18	Ok	5.00	0.03	2.70	0.75	Ok
Cognition	2.94	0.09	4.15	0.53	Ok	1.82	0.18	2.47	0.78	Ok
Suicidal ideas	0.5	0.82	12.04	0.03	Ok	0.41	0.52	2.36	0.80	Ok
Mood instability	0.07	0.79	3.36	0.50	Ok	0.19	0.66	4.32	0.50	Ok
Irritability	1.00	0.32	4.60	0.33	Ok	9.87	0.00*	4.10	0.54	Flag

Differential test function (DTF)	Tau^2^: −0.001 (small effect)					Tau^2^: 0.11 (medium effect)				

*^♣^Tests uniform DIF which means that one group systematically scores higher*.

*^†^Tests non-uniform DIF which means that one group scores higher at lower levels of depression but the pattern reverses at higher levels of depression*.

*An item is flagged for differential function if either the Mantel-Haenszel or the Breslow-Day test is significant.

With the DIF assessment by age group, cognition showed both uniform DIF. In the calibration sample, respondents above 45 years of age endorsed cognitive problems more frequently. In the validation sample, respondents above age 45 endorsed irritability more frequently. Both cognition and irritability had large test effects in both samples. See Table 6 for details.

**Table 6:** **DIF by age group**.

Item	Sample 1 (*n* = 500)					Sample 2 (*n* = 500)				
	Mantel–Haenszel χ^2^^♣^	*p*-Value	Breslow-Day^†^	*p-*Value	Combined decision rule*	Mantel–Haenszel χ^2^^♣^	*p*-Value	Breslow-Day^†^	*p*-Value	Combined decision rule*
Sad	1.29	0.26	1.82	0.77	Ok	2.27	0.13	1.65	0.90	Ok
No interest	1.68	0.19	1.98	0.85	Ok	0.24	0.62	2.81	0.73	Ok
Sleep	0.20	0.66	1.68	0.89	Ok	6.36	0.01	2.41	0.79	Ok
Cognition	12.26	<0.001*	3.19	0.67	Flag	3.86	0.05	6.74	0.24	Ok
Suicidal ideas	0.27	0.61	4.63	0.46	Ok	3.82	0.05	5.96	0.31	Ok
Mood instability	6.62	0.01	2.56	0.63	Ok	3.11	0.07	4.23	0.52	Ok
Irritability	8.50	0.004	6.35	0.17	Ok	10.95	<0.001*	2.80	0.73	Flag

Differential test function (DTF)	Tau^2^: 0.29 (large effect)					Tau^2^: 0.21 (large effect)				

*^♣^Tests uniform DIF which means that one group systematically scores higher*.

*^†^Tests non-uniform DIF which means that one group scores higher at lower levels of depression but the pattern reverses at higher levels of depression*.

*An item is flagged for differential function if either the Mantel-Haenszel or the Breslow-Day test is significant.

### Test Reliability

The PSI for the initial 11 symptoms was 0.60 for the calibration sample and 0.58 for the validation sample. For the seven retained symptoms (i.e., sad, no interest, sleep, cognition, suicidal ideas, MI, irritability) the PSIs were 0.38 and 0.35 for the calibration and validation samples, respectively. Cronbach alphas for the 11 symptoms (nine original, plus MI and irritability) were 0.71 and 0.70 for the calibration and validation samples, respectively, and 0.58 for both samples for the seven retained symptoms.

In *post hoc* analysis, we examined the PSI and alpha for the standard nine DSM/ICD symptoms. PSIs were 0.52 and 0.51, and Cronbach alphas were 0.70 and 0.67 for calibration and validation, respectively.

## Discussion

In this work, we sought to clarify what symptoms form the most statistically cohesive set to measure depression as a construct. We now discuss the practical and theoretical implications of our findings.

### Measuring Depression

We found that the nine depression symptoms in the DSM/ICD systems are not unidimensional. In practice, this means that the “5 of 9” rule (or “4 of 10” for ICD) is probably not warranted because the symptoms do not all tap the same attribute. A more homogeneous set of indicators is achieved by removing weight and appetite change, agitation and retardation, and feelings of worthlessness and inappropriate guilt and fatigue from the core of the major depression syndrome. The findings that MI and irritability fit the Rasch model indicates that they belong to the core network/cluster of symptoms that includes sadness and anhedonia (49).

While irritability appears to be endorsed differentially by sex and by age group, MI is invariant with respect to both characteristics. That irritability is identified as a DIF item should not automatically exclude it from the list. One possibility is to adjust for the DIF effect of gender and/or age in assessing the severity of depression (50). This would be difficult to implement in a paper and pencil test, but could be solved by computer adaptive testing that takes covariates into account.

Seven items – sad, lack of interest, sleep, cognition, suicidal ideas, MI, and irritability – could serve as the kernel for a depression measure, but on their own do not reliably separate depressed persons from the rest. Likewise, the list of DSM/ICD symptoms fails the typical criterion for internal consistency (alpha < 0.80) and also falls short of distinguishing the depressed from the rest (PSI < 0.70). Clinical judgment and contextual information may need to be taken into account apart from the canonical list of symptoms. For use outside of the clinic, scales such as the PHQ-9, Beck Depression Inventory, HADS, CES-D, and the like are only recently being examined using item response theory (51–53).

### Reconceptualizing Depression

Mood instability is common in depression (23, 25) and has been shown to be a precursor of depression (24). Our current results provide evidence that MI is a symptom of depression. According to DSM, relatively short durations of MI phases would not meet episode criteria for major depression (2 weeks) or for hypomania (4 days) (4). If the patient reports rapid mood fluctuations, it is typical to either dismiss MI as clinically unimportant or to concurrently diagnose a personality disorder, particularly borderline personality disorder where both MI and irritability are DSM-V criteria (4). Unfortunately, people who do not fulfill duration criteria may also be considered “well” or at least not depressed and receive no treatment (54), but these people are at higher risk of developing future depression (55). The evidence indicates that intense and frequent mood swings are associated with severe distress (56, 57), and there is merely a quantitative difference between the mood fluctuations of normal individuals and those of patients (58). MI is linked to other indicators of distress and impairment such as health care utilization, medication use, and suicidal thoughts (25, 59) and has recently been proposed to fit the characteristics of the Research Domain Criteria (60).

Irritability is associated with emotional lability in patients with unipolar depression (30) and in university students (30, 61) and is common in depression (29, 30). Mixed depression, which may be defined as “an overlapping of manic and depressive symptoms” includes irritability and emotional lability among its symptoms (62). This presentation is characterized by psychic and motor agitation, accompanied by intense suffering, which put the patient at increased risk of suicide (54). Irritability could be a core symptom of depression (29), an indicator of a more severe and chronic course (30, 63) or a feature of bipolar disorders (64). DSM-V has included irritability as a core symptom of mania, generalized anxiety disorder, and borderline personality disorder. but excluded it as a symptom of major depression. It has previously been rejected as a symptom of major depression as it does not appreciably increase the prevalence above that of sadness and loss of interest (29, 65). Increased prevalence is not necessarily a good basis for defining a syndrome. Conversely, irritability, along with MI, could lead to longstanding interpersonal and adjustment difficulties that could lead to depression (30, 49). In summary, both MI and irritability are observed in a range of psychiatric disorders.

It is uncertain whether agitation and retardation are specific distinguishing features of major depression, melancholia, mixed mood states, atypical depression, bipolar II depression, or anxiety comorbid with mood disorders (66, 67). Agitation is a defining characteristic of a proposed mixed depressive state that has both melancholic and excitatory features, but which does not have the levity in mood of hypomanic patients (62). Our finding is more consistent with a major depression study that reported that agitation could be dropped from the definition of major depression with no loss of validity (68).

The Feighner symptom of “self-reproach or guilt” was expanded in DSM-III to include “feelings of worthlessness …” as part of the DSM trend to broaden the criteria for major depression (69). There is a clear semantic difference between guilt (worry) about past misdeeds and anxiety (worry) about future threats and perhaps feeling helpless, but this distinction might not be meaningful for people with common mental disorders, high comorbidity, or high distress (70–73). Our results replicate findings of a previous Rasch analysis of the PHQ-9 scale that guilt was not coherent with the model of depression (51).

We eliminated fatigue because of higher than expected correlation with cognitive problems. This could be the result of similar wording: both symptoms are presented as diminished ability. An alternative to eliminating this item is rewording either fatigue or cognitive problems. Retaining this item is probably the more prudent course of action. Although one study reported that fatigue is not unidimensional with the other depression symptoms (74), several other studies reported that it satisfies the Rasch model (11, 52, 75).

It should be emphasized that the misfitting items are frequently experienced by patients with major depression. What is in question is whether they are central to the network of symptoms comprising the depressive syndrome and whether they are useful in its assessment. The search for underlying biological or psychological aberrations (76) or treatment (77) for depression is probably hampered by a heterogeneous cluster of symptoms.

Our study has several limitations. First, the data are based on retrospective recall with all of the disadvantages of this method (78). People who are depressed have a general negative recall bias that might affect reporting of symptoms (78). Second, the CIS-R was designed to elicit the ICD-10 criteria for depression, although the symptoms are very similar to those of DSM-V. Third, we did not consider duration criteria for the individual symptoms, and thus cannot be certain that all symptoms occurred at the same time. However, all duration criteria of major depression and similar groupings are arbitrary and, theoretically, symptoms may occur sequentially and still indicate the same syndrome (55, 79). Fourth, we studied MI and irritability, but other common symptoms such as anxiety, rumination and physical symptoms should be studied (73, 80). Fifth, we performed DIF analysis only by age and gender in a British sample, so further analysis is required to determine if MI and irritability are part of the syndrome across cultures. The differential item status of sleep, irritability, and MI should be addressed by future work. Sixth, while MI and irritability were shown to load on a single factor with depression, we did not have an independent external criterion to serve as a reference. Finally, the PMS did not assess bipolar disorder so it is possible that some of the respondents had bipolar, instead of unipolar depression. We do not think this limitation undermines our findings because MI is a feature of a wide range of psychiatric disorders (22, 81) and, second, the prevalence of people with bipolar depression in the sample in comparison to unipolar depression is likely to have been small. It would be beneficial in replication studies to correlate the scores of patients in our proposed 7-item scale to standard psychometric questionnaires, such as the Mood Disorders Questionnaire or the Affective Lability Scale.

A strength of our study is that it was based on empirical data obtained from an epidemiological sample of the population. Accordingly, it was not constrained by a pre-selected sample with major depression as diagnosed by the criteria being studied.

## Conclusion

Mood instability and irritability are candidate core symptoms of the depressive syndrome and should be part of its clinical assessment.

## Author Contributions

LB, RB, MB, and SM conceptualized the study. SM and LB acquired the data. LB, RB, MB, and SM searched the literature on major depression, irritability, and mood instability. LB analyzed the data. LB and RB wrote the initial draft. MB and SM critically reviewed and commented on the initial draft. LB, RB, MB, and SM interpreted the results. LB, RB, MB, and SM approved the final draft for submission and are accountable for the accuracy and integrity of the work.

## Conflict of Interest Statement

The authors declare that the research was conducted in the absence of any commercial or financial relationships that could be construed as a potential conflict of interest.
