# Supplementary material for: Mood Instability and Irritability as Core Symptoms of Major Depression: An Exploration Using Rasch Analysis
Source: Front Psychiatry. 2016 Oct 26;7:174. doi: 10.3389/fpsyt.2016.00174 (PMC5080527; doi:10.3389/fpsyt.2016.00174)
Supplement: Supplementary file 1 [file data_sheet_1.docx]

# **8** **Appendix**

## Analysis steps

### Step 0: Sampling

Two competing demands were considered in the choice of sample size: obtaining precise parameter estimates (which requires large samples) and controlling Type 1 error (which increases with sample size) (1). On the one hand, chi-square tests (which assess goodness of fit), depend on a limiting distribution that is reached when the sample size is 500, for a test with more than 10 questions (2). On the other hand, detecting misfitting items is more likely with larger samples (3). In fact, real-world data is guaranteed to be inconsistent with the Rasch model simply because of large sample size (4). With these caveats in mind, we decided to draw two random samples of 500 respondents from the dataset. The first sample was used for calibration and the second, for validation. Subjects that either endorsed all 11 or zero candidate items were not sampled because they are not useful in estimating the Rasch parameters (1). Using split samples (Step 0) and then testing for dimensionality (Step 1, next paragraph) replicates the approach of an earlier study (5).

### Step 1: Test of dimensionality

The objective of this step was to assess whether depression symptoms can be accounted for by a single latent dimension or factor. Although dimensionality can be assessed as part of fitting the Rasch model (6), it was reasonable to do an exploratory factor analysis first (7) because mood instability and irritability are not included in the ICD and DSM lists. Moreover, dimensionality testing before performing Rasch analysis was justified by Ponocny (8). Horn’s parallel analysis was used to examine dimensionality. This procedure is a modification of the factor analytic rule to retain the number of factors for which the eigenvalue is greater than one. Horn indicated that in a finite population, eigenvalues greater than one are possible due to noise (9). We required that the eigenvalue be greater than the 95^th^ percentile of the eigenvalues of 5000 simulated datasets having the same number of variables and cases—in other words, datasets with noise only. The analysis was performed using the *paran* package in R (10, 11).

### Step 2: Fitting the Rasch model

In this step, we tested whether the 11 items are consistent with a probabilistic Guttman pattern. Using the high jump analogy once more, the Guttman pattern holds when a jumper clears a given height *h* and everything lower, but fails to clear heights greater than *h*. This needs to be true of all jumpers, allowing for a different *h* for each jumper. The Guttman pattern is violated when for example, a jumper clears 7 feet but then fails to clear 6 feet. The Guttman pattern is recognizable as a stair-case like shape where symptoms are ranked from “easiest” (most endorsed) to “most difficult” (least endorsed) on the horizontal axis, and the persons are ranked from most to least depressed on the vertical axis. The term *probabilistic* is used because the Rasch model allows for some amount of noise.

In examining each item’s fit with the Rasch model, we inspected mean square statistics. Following the recommendation to take sample size into account (12), we required that infit mean square values be between 0.91 and 1.09. When the data fit the Rasch model perfectly, the mean square values for each item are 1.0. Rasch modeling was implemented in the *TAM* package (13) in R.

### Step 3: Test for local independence

The Rasch model postulates that after accounting for the latent factor, the response to a particular symptom should be independent of responses to other questions. What this means in practice is that when a particular individual’s true depression level is already known, knowing the response to a particular item should not make the response to another item easier to predict (14). We followed the guidelines that residual correlations should not exceed 0.2 (15) and for the p values (adjusted for multiple comparisons through Holm’s method) to be less than .05 (16). This ensures that factors unrelated to depression itself—such as relative item positions in the list, or very similar wording—are not at work. Local independence was tested using the *sirt* package (17) in R.

### Step 4: Test for differential item function (DIF)

DIF analysis is concerned with possible bias in the endorsement of depression items across respondent characteristics (18). A depression scale that is consistent with the Rasch model should be unrelated to factors such as age and sex, controlling for depression level. Such a scale is said to be an invariant measure. We tested for both uniform and non-uniform DIF by age and sex. Age was dichotomized into <45 and ≥45. Uniform DIF is comparable to main effects ANOVA, in which one group systematically has higher scores at each trait level than the other group while non-uniform DIF is comparable to interaction effects ANOVA (3). Mantel-Haenszel and Breslow-Day tests were used to detect uniform and non-uniform DIF respectively, as implemented in the *difR* package in R (19). An item is flagged using the combined decision rule, i.e. if either of the two tests is significant (20).

In addition to DIF detection, we also examined differential test function (DTF). Unlike DIF which works at the item level, DTF works on the entire test. It is concerned with whether the combined DIFs of all items (if any) have a substantial effect on the entire scale. DTF analysis was implemented in the stand-alone DIFAS program (21).

## Step 5: Test reliability

The internal reliabilities of the original list of 11 items and of the retained items were assessed using Cronbach’s alpha and the person separation index (PSI). PSI assesses the ability of the scale to distinguish different strata of respondents along the latent trait (22, 23). Stated another way, person separation is the proportion of uncertainty about a person’s depression level that is explained by the test (24). A PSI of at least 0.7 is required to distinguish between depressed and non-depressed patients (23).

1. Chen WH, Lenderking W, Jin Y, Wyrwich KW, Gelhorn H, Revicki DA. Is Rasch model analysis applicable in small sample size pilot studies for assessing item characteristics? An example using PROMIS pain behavior item bank data. Qual Life Res. 2014;23(2):485-93.

2. Alexandrowicz RW, Draxler C. Testing the Rasch model with the conditional likelihood ratio test: sample size requirements and bootstrap algorithms. Journal of Statistical Distributions and Applications. 2016;3(2):1-25.

3. Tennant A, Penta M, Tesio L, Grimby G, Thonnard JL, Slade A, et al. Assessing and adjusting for cross-cultural validity of impairment and activity limitation scales through differential item functioning within the framework of the Rasch model: the PRO-ESOR project. Med Care. 2004;42(1 Suppl):I37-48.

4. Martin-Lof P. The notion of redundancy and its use as a quantitative measure of the discrepancy between a statistical hypothesis and a set of observational data. Scandinavian Journal of Statistics. 1974;1:3-18.

5. Olino TM, Yu L, Klein DN, Rohde P, Seeley JR, Pilkonis PA, et al. Measuring depression using item response theory: an examination of three measures of depressive symptomatology. Int J Methods Psychiatr Res. 2012;21(1):76-85.

6. Linacre JM. Rasch analysis first or factor analysis first? Rasch Measurement Transactions. 1998;10(3):509-11.

7. Chen WH, McLeod L, Coles T. Rasch First? Factor First. ISPOR 17th Annual European Congress; November 8-12, 2014; Amsterdam, Netherlands2014.

8. Ponocny I. Nonparametric goodness-of-fit tests for the Rasch model. Psychometrika. 2001;66(3):437-59.

9. Horn JL. A Rationale and Test for the Number of Factors in Factor-Analysis. Psychometrika. 1965;30(2):179-85.

10. Dinno A. paran: Horn's Test of Principal Components/Factors. 2012.

11. R Core Team. R: A language and environment for statistical computing. Vienna, Austria: R Foundation for Statistical Computing; 2015.

12. Smith RM, Schumacker RE, Bush MJ. Using Item Mean Squares to Evaluate Fit to the Rasch Model. Journal of Outcome Measurement. 1998;2(1):66-78.

13. Kiefer T, Robitzsch A, Wu M. TAM: Test Analysis Modules. 1.17-0 ed2016.

14. Bartolucci F, Bacci S, Gnaldi M. Statistical analysis of questionnaires : a unified approach based on R and Stata. Boca Raton: CRC Press, Taylor & Francos Group; 2016. xxi, 308 pages p.

15. Chen WH, Thissen D. Local dependence indexes for item pairs: Using item response theory. J Educ Behav Stat. 1997;22(3):265-89.

16. Chen JS, Torre J, Zhang Z. Relative and Absolute Fit Evaluation in Cognitive Diagnosis Modeling. J Educ Meas. 2013;50(2):123-40.

17. Robitzsch A. sirt: Supplementary Item Response Theory Models. version 1.10-0 ed2016.

18. De Ayala RJ. The theory and practice of item response theory. New York: Guilford Press; 2009. xv, 448 p. p.

19. Magis D, Beland S, Raiche G. difR: Collection of methods to detect dichotomous differential item functioning (DIF). version 4.6 ed2015.

20. Penfield RD, Algina J. Applying the Liu-Agresti estimator of the cumulative common odds ratio to DIF detection in polytomous items. J Educ Meas. 2003;40(4):353-70.

21. Penfield RD. DIFAS: Differential item functioning analysis system. Appl Psych Meas. 2005;29(2):150-1.

22. Bond TG, Fox CM. Applying the Rasch model : fundamental measurement in the human sciences. 2nd ed. Mahwah, N.J.: Lawrence Erlbaum Associates Publishers; 2007. 340 p. p.

23. Elhan AH, Kutlay S, Kucukdeveci AA, Cotuk C, Ozturk G, Tesio L, et al. Psychometric properties of the Mini-Mental State Examination in patients with acquired brain injury in Turkey. J Rehabil Med. 2005;37(5):306-11.

24. Adams RJ. Reliability as a measurement design effect. Studies in Educational Evaluation. 2005;31(2-3):162-72.
